# On-Farm Methane Mitigation and Animal Health Assessment of a Commercially Available Tannin Supplement in Organic Dairy Heifers

**DOI:** 10.3390/ani14010009

**Published:** 2023-12-19

**Authors:** Ashley Schilling-Hazlett, Edward J. Raynor, Logan Thompson, Juan Velez, Sara Place, Kim Stackhouse-Lawson

**Affiliations:** 1 CSU AgNext, Department of Animal Sciences, Colorado State University, Fort Collins, CO 80523, USA; 2 Department of Animal Science and Industry, Kansas State University, Manhattan, KS 66502, USA; 3 Aurora Organic Dairy, Boulder, CO 80302, USA

**Keywords:** on-farm research, methane, organic dairy, climate-smart conservation practices, animal health, animal performance

## Abstract

**Simple Summary:**

On-farm research that investigates commercially relevant climate-smart conservation practices is critical in order to develop practical sustainability strategies in agricultural production systems. The purpose of this study was to investigate on-farm methane (CH_4_) mitigation potential and animal health outcomes using a commercially available tannin as a feed additive in dairy heifers reared under an organic production system. Enteric CH_4_ was measured using one GreenFeed and animal health was evaluated via select antioxidant enzyme and oxidate stress biomarker concentrations. No effect was observed for daily enteric CH_4_ production, average daily gain (ADG), dry matter intake (DMI), or oxidative stress biomarker concentration. Tannin supplementation did have an effect on the concentration of the antioxidant-enzymes-reduced glutathione (GSH) and superoxide dismutase (SOD). Future research may consider the investigation of animal health benefits due to tannin supplementation, such as linking the effect of tannins to the improved antioxidant status of heifers, which may be of particular interest in organic dairy production systems. The observations from this study highlighted the challenges for future on-farm sustainability research and the scalability of potential CH_4_ mitigation strategies.

**Abstract:**

The objective of this experiment was to demonstrate the effectiveness of a commercially available tannin product (Silvafeed^®^ ByPro, 70% tannic acid) as an enteric methane (CH_4_) mitigation and preventative animal health strategy in Holstein heifers (BW = 219 ± 17 kg; 9 mo), reared under organic production system requirements. Twenty heifers were randomly assigned to one of four commercial tannin supplementation treatments as follows: 0% (0 g/hd/d; CON), 0.075% (~5 g/hd/d; LOW), 0.15% (~10 g/hd/d; MED), and 0.30% (~21 g/hd/d; HIG) of dry matter intake (DMI). Heifers received their treatment in individual animal feeding stanchions and were fed a basal total mixed ration (TMR) through four SmartFeed Pro intake measurement bunk systems (C-Lock Inc., Rapid City, SD, USA) for 45 d. An automatic head chamber system (AHCS; i.e., GreenFeed, C-Lock Inc., Rapid City, SD, USA) was used to continuously evaluate enteric CH_4_ production. No effect was observed among the treatments for CH_4_ emissions (*p* ≥ 0.55), animal performance (*p* ≥ 0.38), or oxidative stress biomarker concentration (*p* ≥ 0.55). Superoxide dismutase (SOD) and reduced glutathione (GSH) concentrations exhibited a linear response to increasing tannin dose (*p* = 0.003), indicating a potential tannin effect on the antioxidant status of dairy heifers. This observation may encourage future tannin research relating to animal health, which may be of particular interest to organic dairy systems. The results of this study suggest that tannin supplementation at 0%, 0.075%, 0.15%, and 0.30% of DMI, did not alter CH_4_ emissions, animal performance, or oxidative stress biomarker concentration in organic Holstein heifers when assessed under an on-farm research approach. Further, the results of this study affirm the challenges associated with on-farm research and the development of climate-smart strategies that are capable of mitigating climate impacts in less controlled environments under standard working conditions.

## 1. Introduction

The on-farm research and verification of climate-smart conservation practices is necessary to consider when developing mechanisms to aid animal agriculture industries to meet climate goals; however, limited research has been conducted in production settings. Compared to traditional research, on-farm research is more challenging due to the inability to control confounding variables. Nonetheless, on-farm derived research results are important both to understand the efficacy of emission mitigation at scale and for encouraging producers to adopt climate-smart conservation practices as they can demonstrate practicality in production, for example, evaluating the tradeoffs and synergies of mitigating environmental impacts without sacrificing animal wellbeing and performance.

The United States (U.S.) dairy industry is responsible for approximately 1.4% of total U.S. greenhouse gas (GHG) emissions [1]. Methane (CH_4_) from enteric fermentation is a significant component of the dairy industry’s GHG footprint [2]. Approximately 25% of total GHG emissions expressed as carbon dioxide equivalents (CO_2_e) originate from enteric fermentation in dairy cattle production systems [1]. While there has been considerable research conducted to identify the strategies that reduce enteric CH_4_ [3,4,5,6], limited progress has been made implementing these strategies on farms, challenging supply chain GHG reduction and many company NetZero goals.

One extensively evaluated enteric CH_4_ mitigation strategy is tannins [3,4,5,6]. Currently, multiple hypotheses have been postulated regarding the mode of action that tannins utilize to decrease CH_4_ production, such as: (1) tannins act directly on methanogens [7,8,9,10], (2) tannins affect protozoa populations associated with methanogens [11,12], and (3) tannins affect fibrolytic bacteria leading to a decrease in ruminal fiber degradation [13]. Aboagye and Beauchemin [14] suggest that tannins may function via all, some, or any of the proposed mechanisms, because in studies where CH_4_ reductions are reported, there is a variable range in the decrease of CH_4_. While research has been conducted in controlled settings, on-farm research is important to further evaluate the scalability of tannins as a climate-smart mitigation strategy. This production-based research is especially critical as supply chains begin to incentivize the implementation of climate-smart practices and measure, report, and verify the corresponding emissions reductions.

Tannins also have the potential to negatively impact feed intake, digestion and absorption of nutrients, and animal performance measures [15,16,17]. When including condensed tannins in the diet of ruminants at concentrations greater than 55 g per kg dietary dry matter (DM), a reduction in voluntary feed intake, digestibility, and rate of body growth can be the result [15]. Additionally, the effect of tannins can be influenced by a variety of tannin factors such as class, concentration/purity, dose, type, and other factors such as animal species, physiological state of the animal, and diet composition [15,16,17]. Thus, commercially available products have aimed for low-level supplementation to avoid negative animal performance impacts.

Tannins may also act as a preventative animal health strategy due to the evidence of benefits such as antioxidant, anti-microbial, anti-parasitic, anti-inflammatory, and anti-viral effects [18]. A requirement of an animal reared under the U.S. Department of Agriculture (USDA) organic dairy certification requires animals to have not received antibiotics [19]. The investigation of commercially available tannin products may be of particular interest to organic dairy systems since tannins are naturally occurring and could be implemented without violating USDA Organic Dairy requirements as a preventative animal health practice and to reduce GHG emissions. Yet, limited research has been conducted with growing dairy heifers to determine how tannins might impact antioxidant enzyme concentration and oxidative stress, which is when an animal’s cellular ability for antioxidant enzymes to neutralize free radicals is imbalanced due to the accumulation of oxygen reactive species.

The objective of this experiment was to investigate the effectiveness of a commercially available tannin product as an on-farm climate-smart conservation practice to mitigate enteric CH_4_ and improve animal health in growing organic Holstein heifers. We hypothesized that (1) low-level tannin supplementation would not negatively impact animal performance, (2) as tannin dose increased, enteric CH_4_ emissions would decrease, and (3) antioxidant biomarkers would increase as the oxidative stress biomarker decreased.

## 2. Materials and Methods

This study was approved by the Colorado State University (CSU) Institutional Animal Care and Use Committee (#2341) prior to project initiation and was conducted at a commercial certified organic dairy in Gill, Colorado (40.537715, −104.506222) from October 2021 through January 2022 in a drylot pen (~0.06 hectares; 0.003 hectares/hd). The mean annual October–January temperature minimum (1963–2022) is −7.3 °C, with the study period mean of −6.3 °C and daily minimums ranging from −29.4 to 3.9 °C. Minimums during the final week of data collection were 15% below the 59-year mean (−11.6 vs. −13.3 °C).

The USDA organic dairy certification requires cattle to access pasture for no less than 120 d/yr, and cattle must consume a minimum of 30% of their DMI from pasture during the grazing seasons [18]. This study was conducted outside of the grazing season where cattle could be fed in a drylot setting due to the absence of a pasture requirement, offering more control of the diet and measurement of individual animal intake, which is important to assess when investigating enteric CH_4_ emissions.

### 2.1. Experimental Animals and Design

Twenty-four, Holstein heifers (BW = 219 ± 17 kg 1SD; age: 9 mo), reared under organic production system requirements, were acclimated to two SmartFeed Pro trailers (C-Lock Inc., Rapid City, SD, USA) each equipped with two feed intake measurement bunk systems in each trailer, one GreenFeed automated head chamber system (AHCS; C-Lock Inc., Rapid City, SD, USA) and individual animal feeding stanchions. During the 28 d acclimation period, the animals were introduced to the AHCS with entrance alley panels removed as described by Gunter and Bradford [20]. Heifer AHCS visitation was not limited during the acclimation period to motivate voluntary acclimation to the AHCS system. To maintain consistent use of the AHCS during the acclimation period, alley panels were put in place on d 21 and gradually narrowed until one animal at a time could enter and use the AHCS. A 7 d pretrial gas analysis period was conducted from d − to −1 to account for individual animal emissions differences and serve as a baseline for each individual animal in subsequent analyses. Following acclimation, from the 24 acclimated heifers, 20 were selected to participate in the study based on frequency of visitation to the AHCS. Tannin supplementation was evaluated in a dose-response structure. Heifers were blocked by initial body weight and randomly assigned to one of four treatments (CON = tannin supplemented at 0% of dry matter intake (DMI); 0 g/hd/d, LOW = tannin supplemented at 0.075% DMI; ~5 g/hd/d, MED = tannin supplemented at 0.15% DMI; ~10 g/hd/d, and HIG = tannin supplemented at 0.30% DMI; ~21 g/hd/d). Approximate amounts of tannin delivered are represented due to weekly recalculation of the intake and tannin dose which increased throughout the experimental period as heifers grew and intake increased. The commercial tannin product utilized was Silvafeed^®^ ByPro (70% tannic acid), which is a blended chestnut and quebracho tannin product. Supplemental tannin dosage was recommended by the manufacturer and mixed to a total weight of 1 kg with sweet feed to increase the palatability of and bind to the tannin supplement to ensure consumption of the tannin offered. Heifers were fed the tannin supplement corresponding to the treatment dosage in individual animal feeding stanchions daily at 0700 h prior to the delivery of the total mixed ration (TMR) in the SmartFeed Pro feed bunks for 45 d. Heifers were allotted 30 min to consume their assigned treatment, and then were returned to the pen, and orts were weighed. Throughout the duration of the trial, all heifers consumed the entirety of the supplement provided; therefore, no orts were present.

### 2.2. Sample Collection

#### 2.2.1. Enteric CH_4_ Emissions

**Figure 1:**
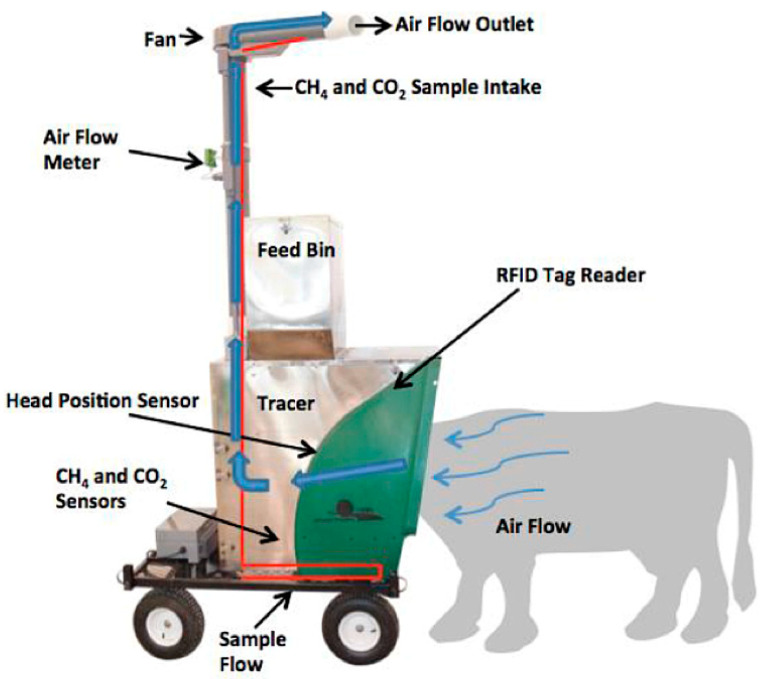
Components of the GreenFeed automated head-chamber system (AHCS) used for measuring daily CH_4_ production from Holstein heifers, adapted from Hristov et al. [20]. The blue arrows represent the air that is pulled from around the animal’s head into the intake manifold and up the pipe, when an animal visits the AHCS. The red line represents a sub-sample of air that is pumped out of the pipe into non-dispersive infra-red sensors for continuous measurement of CH_4_ concentration.

**Figure 2:**
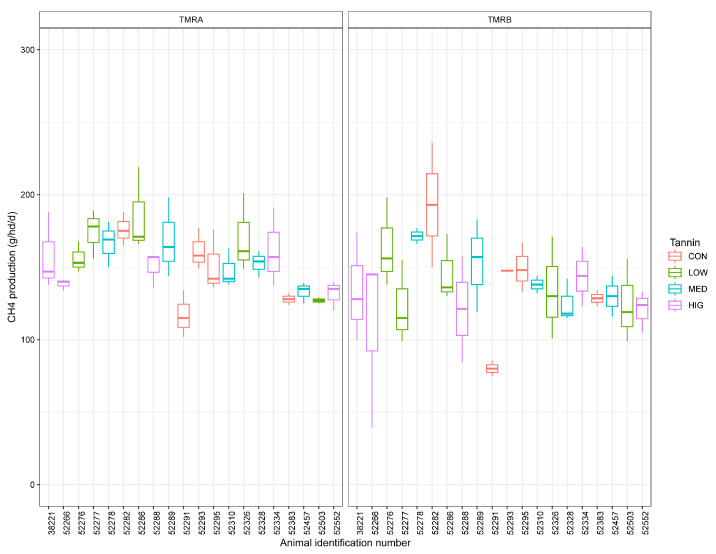
Mean individual daily CH_4_ production from organic Holstein heifers (n = 5 per treatment) in g/hd/d by treatment with tannin supplementation at 0% (CON), 0.075% (LOW), 0.15% (MED), and 0.30% (HIG) of DMI from d 0 to 25 (TMR^A^) and d 26 to 45 (TMR^B^).

Daily enteric CH_4_ production was measured using an AHCS (Figure 1) [20] to determine individual animal CH_4_ emissions, which, as observed in Figure 2, naturally exhibit some degree of variability. Heifers voluntarily utilized the AHCS system, and emissions data were recorded upon recognition of a radio frequency identification (RFID) ear tag placed in the left ear. Organic alfalfa pellets were used in the AHCS as bait feed that was dispensed at each drop during an animal visit. The AHCS was programmed to dispense 6 drops of organic alfalfa pellets as bait at 30 s intervals when an animal was present. This distribution routine encouraged the animal to stay at the AHCS while exhalations were sampled [21]. Only measurements from animals sampled from three to eight minutes were considered sufficient for statistical analysis [22]. Three-minute samples were standardized as the minimum time required to capture at least three eructations during a sampling event, which minimized the variation in CH_4_ emission estimates [23,24,25]. Additionally, airflow through the AHCS is an important factor; therefore, visits reporting airflow below 26 L/s were discarded [22]. Heifers were limited to 4 visits per day, with a minimum 4 h interval between visits. Visit intervals were designed to capture diurnal variation in CH_4_ emissions by encouraging an even distribution of visits throughout a 24 h period [21]. The CH_4_ sensors were calibrated weekly by a manufacturer-installed automatic calibration system (C-Lock Inc., Rapid City, SD, USA). Two CO_2_ recoveries were performed (d −8 and 45) to gravimetrically calibrate the air flux sensor by releasing CO_2_ into the AHCS with a 90 g prefilled CO_2_ cylinder supplied by the manufacturer. During each CO_2_ recovery, three release intervals were conducted where approximately 30 g of CO_2_ was released into the AHCS, with a recovery percentage of ±5%. Each release interval was three minutes in duration with a three-minute waiting period between each release interval. Gravimetric release was compared with the AHCS calculated capture using an internet portal. A clean air filter was fitted to the AHCS when air flow was <33.1 L/s.

#### 2.2.2. Animal Feed Intake

Daily feed intake was measured using four SmartFeed Pro intake measurement bunk systems (C-Lock Inc., Rapid City, SD, USA). Heifers voluntarily utilized the SmartFeed Pro feed bunks, and data were recorded upon recognition of a RFID ear tag placed in the left ear. A TMR was delivered into the SmartFeed Pro bunks daily following treatment feeding at 0700 h.

#### 2.2.3. Feed Samples

Two TMR formulations were fed during the duration of the study. The TMR formulation changed on d 26 of the study as directed by the commercial organic dairy nutrition protocols. The TMR formulations were classified as TMR^A^, a growing heifer ration, consisting of 57.4% roughage and 38.9% concentrate, and TMR^B^, a pre-breeding ration, consisting of 87.4% roughage and 12.1% concentrate (Table 1).

Fresh and residual TMR samples were collected daily from each SmartFeed Pro feed bunk and preserved in WhirlPak (Filtration Group, Madison, Wisconsin, WI, USA) sampling bags at −20 °C until the completion of the study. At study completion, samples were thawed, and dried at 65 °C for 72 h to determine analytic DM (dry matter). Dried samples were then ground to pass a 1 mm screen (Wiley Mill, Model 4; Arthur H. Thomas Co., Philadelphia, PA, USA). Following grinding, the samples were composited by week. Weekly composites were then composited into one sample per TMR formulation and sent to a commercial laboratory (Dairy One Inc., Ithaca, NY, USA) for nutritive analysis (Table 2).

Samples of the commercial tannin product were taken on d 0, 23, and 45, weighed and dried at 65 °C for 72 h to determine analytic DM, composited, and sent to a commercial laboratory (Dairy One Inc., Ithaca, NY) for nutritive analysis (Table 2). Organic alfalfa pellets and sweet feed were collected on d 0, 23, and 45, weighed, and dried at 65 °C for 72 h to determine analytic DM. Dried samples were ground to pass a 1 mm screen (Wiley Mill, Model 4; Arthur H. Thomas Co., Philadelphia, PA, USA). Following grinding, the samples were composited and sent to a commercial laboratory (Dairy One Inc., Ithaca, NY, USA) for nutritive analysis (Table 2).

#### 2.2.4. Animal Body Weights

Heifers were weighed (unshrunk) on d −15, 0, 8, 15, 23, 29, 36, 43, and 45 before treatment feeding at 0700 h on a validated scale (Tru-Test AP600 platform and Tru-Test ID5000 scale indicator) using the methodologies described by Thompson et al. [26]. The average daily gain (ADG) was calculated using weekly weights collected from d 0 to 45 via linear regression.

#### 2.2.5. Blood Samples

Malondialdehyde (MDA), a product of lipid peroxidation, was used as an indicator of oxidative stress [27]. Antioxidant enzyme activity was assessed through the analysis of superoxide dismutase (SOD) and reduced glutathione (GSH) concentrations in serum due to historic research using these two enzymes and the role they play in antioxidative status, despite several endogenous antioxidant enzymes having the ability to convert oxygen-derived free radicals into less dangerous forms [28]. Moreover, blood urea nitrogen (BUN) and creatinine were assessed as additional blood parameters used to evaluate N impacts of tannin supplementation.

Blood was collected in two BD Vacutainer^®^ blood collection tubes (Fisher Scientific, Pittsburgh, PA, USA) from the jugular vein on d 0, 23, and 45 between 0700 h and 0900 h, placed on ice, and transported to CSU laboratory facilities. Blood was centrifuged in a Thermo IEC Centra^®^ GP8 Centrifuge for 12 min at 3400 RPM. Following centrifuging, serum was pipetted into Eppendorf Tubes^®^ (Eppendorf AG, Hamburg, Germany) and stored at −20 °C. Following study completion, serum was used to draw inferences on oxidative stress by deriving MDA, SOD, and GSH concentrations using the TBARS Assay Kit, Superoxide Dismutase Assay Kit, and Glutathione Assay Kit, respectively (Cayman Chemical Company, Ann Arbor, MI, USA). The TBARS Assay Kit is a colorimetric assay used to determine MDA concentration which is a commonly used biomarker for oxidative stress. The Superoxide Dismutase Assay Kit is a fluorometric analysis that analyzes SODs, which are crucial components in the cellular antioxidant defense mechanism [29]. The Glutathione Assay Kit is a fluorometric analysis used to analyze GSH. Glutathione occurs in two states in healthy cells, GSH and oxidized glutathione (GSSG), with the majority being in the GSH form. Glutathione was evaluated since it is a well-established antioxidant in cells and plays a critical role in the maintenance and regulation of proper physiological functioning [30].

### 2.3. Statistical Analysis

JMP^®^ Pro (JMP^®^, v. 16.2.0. SAS Institute Inc., Cary, NC, USA, 1989–2021) software was used for initial data visualization and correlation analyses. Subsequent analysis of data was conducted using R© (R Core Team, 2021, v. 4.1.2) software. The data were analyzed using the gls() function within the R package nlme in which tannin treatment, TMR diet, treatment × TMR diet, and mean 7 d pre-trial CH4 (g/d) baseline were fixed factors, and an individual animal was a random intercept. A first-order autoregressive error structure was also included for repeated measures on each animal. The covariance structure that best fit the data was selected according to Schwartz’s Bayesian information criterion [31]. The model diagnostics included testing for normal distribution of the error residuals and homogeneity of variance. The assumptions were adequately held. Dependent variables were ADG, DMI, G:F (kg BW gain/kg DMI), daily CH4 production (g CH4/hd/d), CH4 as a percentage of gross energy (GE) intake (Ym), CH4 yield (MY; g CH4/kg DMI), CH4 emission intensity (EI; g of CH4/kg ADG), BUN (mg/dL), creatinine (mg/dL), MDA (μM), SOD (units/mL), and GSH (μM). Daily CH4 production was analyzed using the 7 d pretrial emissions rate as a covariate (i.e., an individual-specific baseline measurement of pretrial emissions) [20]. The average daily gain was determined via the slope coefficient of a linear regression as a function of gain and day using lm() (R Core Team, 2021, v. 4.1.2) using weights collected from d 0 to 45 [32]. The effect of treatment was determined to be significant at α < 0.05 and a tendency between 0.05 < *p* ≤ 0.10. Sattherwaite’s approximation was used to calculate the effective degrees of freedom of a linear combination of independent sample variances. To examine a potential dose-response relationship for heifers consuming increasing levels of tannin, linear and quadratic effects were assessed using orthogonal contrasts [33].

## 3. Results

The net quantity of tannin intake, based on observed DMI, was CON = 0 g/hd/d, LOW = 5 g/hd/d, MED = 10 g/hd/d, and HIG = 21 g/hd/d.

### 3.1. Methane Emissions Parameters

Heifer nutrition was managed in accordance with on-farm protocols resulting in a dietary change from TMR^A^ to TMR^B^. Enteric CH_4_ production was reduced from TMR^A^ to TMR^B^ (*p* = 0.002; Table 3). However, the TMR transition did not impact the response at any tannin concentration level (treatment × diet; *p* ≥ 0.52). Daily CH_4_ production did not differ between heifers not receiving supplemental tannin (control) and those receiving supplemental tannin (*p* = 0.88). Control heifers produced on average 146 g of CH_4_/hd/d, whereas heifers receiving supplemental tannin produced on average 145 g of CH_4_/hd/d. Similarly, contrasts did not show an effect of increasing treatment level (linear, *p* = 0.56; quadratic, *p* = 0.52). Further, responses of additional emissions metrics did not differ between control heifers and heifers consuming tannin: Y_m_ (*p* = 0.0.92), MY (*p* = 0.91), or EI (*p* = 0.55). When considering the effect of increasing tannin dose, the responses were similar to the control for Y_m_ (linear, *p* = 0.13; quadratic, *p* = 0.21), MY (linear, *p* = 0.13; quadratic, *p* = 0.21), or EI (linear, *p* = 0.92; quadratic, *p* = 0.62).

### 3.2. Animal Performance Parameters

Control heifers and heifers consuming tannin showed similar ADG (*p* = 0.38; Table 4). Control heifers gained on average 0.37 kg/d, whereas heifers receiving supplemental tannin gained on average 0.43 g/d. While heifers receiving supplemental tannin had a greater final body weight (FBW) compared to control heifers, a difference was not observed in the final body weight (FBW; *p* = 0.11) but tended to exhibit a quadratic response to dose (quadratic, *p* = 0.07) with a peak being observed in regard to the LOW treatment. Similarly, total DMI did not differ between control heifers and heifers receiving supplemental tannin treatments (*p* = 0.61). Basal TMR DMI was greater for heifers consuming tannin but was not significantly different from that of control heifers (*p* = 0.66). GreenFeed alfalfa pellet bait DMI tended to be greater for heifers receiving supplemental tannin (*p* = 0.07) but did not exhibit a linear (*p* = 0.46) or quadratic (*p* = 0.27) response to dose. The gain to feed ratio (G:F) was similar among control heifers and heifers receiving supplemental tannin (*p* = 0.66). Linear and quadratic contrasts showed no effect for ADG (linear, *p* = 0.90; quadratic, *p* = 0.60), DMI (linear, *p* = 0.42; quadratic, *p* = 0.96), TMR (linear, *p* = 0.45; quadratic, *p* = 0.93), or G:F (linear, *p* = 0.46; quadratic, *p* = 0.94). The diet transition did not impact the response of animal performance at any treatment level (treatment × diet; *p* ≥ 0.35).

### 3.3. Blood Parameters

Oxidative stress was measured via MDA concentration and did not differ between control heifers and heifers receiving tannin supplementation (*p* = 0.55) or diet (*p* = 0.34; Table 5). Linear and quadratic contrasts showed no effect of increasing tannin dose for MDA (linear contrast, *p* = 0.23; quadratic contrast, *p* = 0.87). Antioxidant defense enzymes that were measured included SOD and GSH. A marginal difference in SOD was observed among control heifers and heifers receiving supplemental tannin (*p* = 0.11), with control heifers having a greater SOD concentration. A linear decline in SOD with increasing tannin dose was observed (linear contrast, *p* = 0.01). An effect of tannin consumption was observed between control heifers and heifers receiving tannin supplementation for GSH (*p* = 0.003), where a linear increase with increasing levels of tannin dose was exhibited (linear contrast, *p* = 0.003). The TMR was significant for SOD (*p* = 0.02), but not significant for GSH (*p* = 0.80). The diet transition did not impact the blood parameter response at any treatment level (treatment × diet; *p* ≥ 0.72).

## 4. Discussion

A considerable amount of research detailing the impact of tannin supplementation in controlled research settings exists, but on-farm applications using tannins as a CH_4_ mitigation strategy is lacking [9,10,11,12,13,14,15,33,34,35]. On-farm research is critical for evaluating the scalability of climate-smart conservation practices at the nexus of the three domains of sustainability (economic, environmental, and social). Further, building knowledge and innovation through on-farm research in collaboration with producers serves as a powerful tool when addressing the key challenges associated with the producer adoption of climate-smart strategies, which are the uncertainty of strategy efficacy and quantification of impacts on animal productivity and efficiency [36].

The present study observed that supplementing a commercially available tannin product up to 0.3% of DMI failed to reduce CH_4_ emissions. It was hypothesized that with increasing tannin supplementation at a low dose, CH_4_ emissions from dairy heifers would be reduced, while avoiding the potential negative performance impacts that have been observed [15,16,17]. While DMI was not observed to be impacted by supplemental tannin dose, CH_4_ emissions from heifers receiving tannins did not decrease when compared to control heifers at any inclusion level. It is known that variability in the ability for tannins to decrease CH_4_ emissions can be attributed to numerous animal biological factors and tannin factors [14,15,16,17]. Similar CH_4_ results have been reported in preexisting literature assessing tannin supplementation as a CH_4_ mitigation strategy [33,34], and it is likely that the level of supplementation was below the threshold capable of altering the microbiological factors in the rumen that could contribute to a decrease in CH_4_ [14]. However, the doses used were suggested by the company and are important to follow in on-farm research.

As evident from the present study, conducting on-farm research does not come without challenges. It is known that diet formulation will impact the efficacy of tannins as an enteric CH_4_ mitigation strategy, but operational nutrition protocols for the partnering farm dictated a change in the plane of nutrition as growing heifers prepared for first breeding. This challenge highlights the difficulty in measuring, reporting, and verifying GHG mitigation practices on farms. These findings reiterate the obstacles to developing climate-smart conservation practices, which is the requirement for persistent CH_4_ emissions reductions, even across changes in nutritional inputs. In future research this will be challenging to overcome since it is known that nutritional inputs impact CH_4_ production in ruminant livestock [37], and yet diets are constantly changing in production settings. Under this on-farm approach, diet had a significant impact on CH_4_ production, which is consistent with preexisting observations [37]. However, this study found CH_4_ production to be 22.4% greater when heifers were fed TMR^A^, compared to TMR^B^, which contradicts preexisting observations that higher roughage diets result in higher CH_4_ production [37]. We postulate that the extreme winter weather conditions prompting the onset of cold stress while heifers were on TMR^B^ may have influenced their CH_4_ emissions. However, the present study did not observe a diet by treatment interaction or a treatment effect. Future research may be necessary to assess the replicability of the results observed under the present on-farm approach.

An underlying theme of climate-smart conservation practices, as they relate to animal agriculture, is the ability to support food security, which is influenced by animal productivity [38]. In the present study, animal performance was quantified via ADG, DMI, and G:F. Diet had a significant impact on animal performance parameters. This observation is logical considering that TMR^A^ was intended for slower growth of heifers prior to first breeding. This observation is affirmed by extensive knowledge that nutrition influences animal performance [39]. An observed unintended consequence of feeding tannins in past research has been a decrease in DMI [15,16,17]. In the present study, DMI did not significantly differ among the treatments but was numerically greater for heifers receiving supplemental tannin when compared to control heifers. This observation is a positive outcome, considering that animal performance is not inhibited by tannins, as hypothesized due to the low dose of supplemental tannin being tested. These findings are consistent with studies conducted with similar objectives [33,34]. Additionally, while not significant, heifers receiving tannin supplementation exhibited greater ADG when compared to control heifers. Greater ADG exhibited by heifers receiving supplemental tannin could be attributed to greater DMI observed and/or other animal physiological, biological, and metabolic factors. In future research, increasing tannin dose may be warranted to effectively decrease CH_4_ emissions, but should still target avoiding negative animal performance consequences.

Animal health and wellbeing can also be impacted directly and indirectly by a changing climate and should be considered when developing climate-smart strategies [40]. Here, the antioxidant enzyme, SOD, was impacted by the diet, but no treatment by diet interaction was observed. Additionally, SOD was observed to linearly decline as treatment dose increased, with SOD concentration being the lowest for the HIG treatment. Moreover, GSH exhibited a linear increase as treatment dose increased. When considering these observations, GSH responded in a manner consistent with the study hypothesis, while SOD did not. The response that GSH exhibited to tannin supplementation has been previously reported [41]. Future research may further evaluate the antioxidant characteristics of tannins and other biological factors that share commonalities. Due to tannins being a naturally occurring plant secondary compound, with observed antioxidant characteristics, there may be an opportunity for tannins to be used as a preventative animal health measure in organic dairy systems where antibiotic use is restricted. This observation generates a need for future work that quantifies health and welfare in addition to performance when conducting research to address climate-smart conservation goals.

Recently, funding opportunities have challenged researchers to investigate climate-smart strategies for reducing emissions from agricultural systems using on-farm research. Benefits of on-farm research cannot be overlooked, such as (1) demonstrating scalable potential and strategy effectiveness in production environments, (2) increasing collaboration among multiple stakeholder groups, and (3) direct dissemination of findings through peer-to-peer producer groups who will put strategies into practice. However, the increasing challenges associated with conducting on-farm research are often overlooked. Not only is the repeatability of on-farm research a challenge due to differences in production systems, but also there is a cost, not only of the physical research activities, but an indirect cost placed upon the producer and partnering organization. When considering research costs, the measurement equipment alone can cost hundreds of thousands of dollars, ultimately limiting the quantification of individual animal feed intake and methane measurements. Due to these challenges, it is likely that statistical power will always be challenged, and measurement, reporting, and verification of emission reductions will be limited.

## 5. Conclusions

On-farm testing of potential mitigation strategies is important in order to incentivize producer adoption and assess the scalability and effectiveness of proposed CH_4_ mitigation strategies. The results of this study suggest that tannin supplementation at 0%, 0.075%, 0.15%, and 0.30% of DMI, did not mitigate CH_4_ emissions, did not negatively impact animal performance, and did not alter oxidative stress biomarker concentration in Holstein heifers, reared under an organic production system. However, observations support that supplemental tannin dose did alter the concentration of antioxidant enzymes, GSH, and SOD. Moreover, the observations also dictate that nutrition management may serve as a barrier to evaluating the effectiveness of potential climate-smart conservation practices when studied on farms. Further investigations should be conducted to determine the repeatability of the results in production environments.

## Figures and Tables

**Table 1:** Diet composition of organic TMR on a dry matter basis fed in northeastern Colorado from November 2021 to January 2022.

Diet Composition ^1^		
Ingredients	TMR^A^	TMR^B^
Alfalfa Hay, %	4.8	36.8
Oat Hay, %	9.1	9.8
Straw, %	4.4	0.0
Haylage, %	15.2	23.0
Corn Silage, %	23.9	17.8
Mixed Grains, %	30.6	7.4
Ground Corn, %	8.3	4.7
Minerals, %	3.8	0.56

^1^ Total mixed ration (TMR), TMR^A^ = composite samples of TMR fed from d 0 to 25, TMR^B^ = composite samples of TMR fed from d 26 to 45.

**Table 2:** Nutritive analysis of organic dietary constituents on a dry matter basis fed in northeastern Colorado from November 2021 to January 2022.

	Feed Nutritive Analysis ^1^				
Item ^2^	TMR^A^	TMR^B^	AP	SF	TAN
DM, %	45.8	45.0	92.0	93.0	91.6
CP, %	19.0	18.6	20.0	21.1	1.8
TDN, %	72	70	61	81	90
GE, cal/g	4571	4603	4668	4841	5341
% ADF ^3^	20.9	27.7	30.8	7.6	0.2
% NDF ^4^	29.7	36.7	35	17.6	0.3

^1^ Total mixed ration (TMR), TMR^A^ = composite samples of TMR fed from d 0 to 25, TMR^B^ = composite samples of TMR fed from d 26 to 45, AP = composite sample of organic alfalfa pellets, SF = composite sample of sweet feed, and TAN = composite sample of tannin supplement. ^2^ Dry matter (DM), crude protein (CP), total digestible nutrients (TDN), gross energy (GE), acid detergent fiber (ADF), and neutral detergent fiber (NDF). ^3^ ADF was determined via wet chemistry. ^4^ Amylase and sodium sulfite treated NDF was determined via wet chemistry.

**Table 3:** Mean CH_4_ emissions measurements for heifers supplemented with Silvafeed^®^ ByPro from d 0 to 45 fed in northeastern Colorado from November 2021 to January 2022.

	Control	Tannin	SE	*p*-Value ^1^	Treatment ^2^				SE	*p*-Value ^3^		
Parameter ^4^					CON	LOW	MED	HIG		Linear	Quadratic	TMR
n	5	15			5	5	5	5				
CH_4_ Production, g CH_4_/hd/d	146	145	5.2	0.88	146	145	149	142	5.2	0.56	0.52	0.002
Y_m_, g CH_4_ % GE intake	6.7	6.8	0.01	0.92	6.7	7.7	6.8	5.7	0.01	0.13	0.21	0.01
MY, g CH_4_/kg DMI	23.4	23.6	2.2	0.91	23.4	27.1	23.9	19.9	2.2	0.12	0.21	0.01
EI, g CH_4_/kg ADG	430	386	61.7	0.55	430	338	412	407	13.7	0.94	0.62	0.004

^1^ Significance *p* < 0.05. ^2^ Treatments for tannin supplementation at 0% (CON), 0.075% (LOW), 0.15% (MED), and 0.30% (HIG) of DMI. ^3^ Significance *p* < 0.05. ^4^ Methane (CH_4_) production, average daily gain (ADG), dry matter intake (DMI), CH_4_ as a % of GE intake (Y_m_), CH_4_ yield (MY), CH_4_ emissions intensity (EI), feed efficiency (G:F).

**Table 4:** Mean performance measurements for heifers supplemented with Silvafeed^®^ ByPro from d 0 to 45 fed in northeastern Colorado from November 2021 to January 2022.

	Control	Tannin	SE	*p*-Value ^1^	Treatment ^2^				SE	*p*-Value ^3^		
Parameter ^4^					CON	LOW	MED	HIG		Linear	Quadratic	TMR
n	5	15			5	5	5	5				
ADG, kg BW gain/d	0.37	0.43	0.07	0.38	0.37	0.49	0.39	0.41	0.01	0.90	0.60	<0.001
FBW, kg	250	252	1.13	0.11	250	254	251	251	1.13	0.70	0.07	-
DMI, kg DMI/d	6.2	6.7	0.72	0.61	6.2	6.4	6.6	7.0	0.72	0.42	0.96	0.06
TMR, kg TMR DMI/d	4.9	5.3	0.72	0.66	4.9	5.0	5.2	5.6	0.72	0.45	0.93	0.07
SF, kg SF DMI/d	0.93	0.93	0.00	-	0.93	0.93	0.93	0.93	0.00	-	-	-
GF, kg GF DMI/d	0.34	0.40	0.03	0.07	0.34	0.44	0.38	0.40	0.03	0.46	0.27	0.02
G:F, ADG/DMI	0.10	0.09	0.03	0.66	0.10	0.11	0.08	0.08	0.03	0.46	0.94	<0.001

^1^ Significance *p* < 0.05. ^2^ Treatments for tannin supplementation at 0% (CON), 0.075% (LOW), 0.15% (MED), and 0.30% (HIG) of DMI. ^3^ Significance *p* < 0.05. ^4^ Average daily gain (ADG), final body weight (FBW), dry matter intake (DMI), total mixed ration (TMR), sweet feed (SF), GreenFeed bait pellets (GF), CH_4_ emissions intensity (EI), feed efficiency (G:F).

**Table 5:** Mean concentration of common oxidative stress and antioxidant enzyme biomarkers derived from serum taken on d 0, 23, and 45 for heifers supplemented with Silvafeed^®^ ByPro from d 0 to 45 fed in northeastern Colorado from November 2021 to January 2022.

	Control	Tannin	SE	*p*-Value ^1^	Treatment ^2^				SE	*p*-Value ^3^		
Parameter ^4^					CON	LOW	MED	HIG		Linear	Quadratic	TMR
n	5	15			5	5	5	5				
MDA, μM	0.51	0.63	0.22	0.55	0.51	0.41	0.75	0.75	0.19	0.23	0.87	0.34
SOD, units/mL	58.18	51.59	4.01	0.11	58.18	51.07	60.85	42.84	3.53	0.01	0.11	0.02
GSH, μM	7.13	10.49	1.07	0.003	7.13	8.80	11.7	10.9	0.94	0.003	0.04	0.80

^1^ Significance *p* < 0.05. ^2^ Treatments for tannin supplementation at 0% (CON), 0.075% (LOW), 0.15% (MED), and 0.30% (HIG) of DMI. ^3^ Significance *p* < 0.05. ^4^ Malondialdehyde (MDA), superoxide dismutase (SOD), and reduced glutathione (GSH).

## Data Availability

Data are available upon request to the corresponding author.

## References

[1] EPA (2021). Inventory of U.S. Greenhouse Gas Emissions and Sinks: 1990–2019 (No. EPA-430-R-21-005).

[2] Rotz A., Stout R., Leytem A., Feyereisen G., Waldrip H., Thoma G., Holly M., Bjorneberg D., Baker J., Vadas P. (2021). Environmental assessment of United States dairy farms. J. Clean. Prod..

[3] Beauchemin K.A., Kreuzer M., O’Mara F., McAllister T.A. (2008). Nutritional Management for enteric methane abatement: A review. Aust. J. Exp. Agric..

[4] Place S.E., Mitloehner F.M. (2010). Invited review: Contemporary environmental issues: A review of the dairy industry’s role in climate change and air quality and the potential of mitigation through improved production efficiency. J. Dairy. Sci..

[5] Hristov A.N., Oh J., Firkins J.L., Dijkstra J., Kebreab E., Waghorn G., Makkar H.P., Adesogan A.T., Yang W., Lee C. (2013). Special topics—Mitigation of methane and nitrous oxide emissions from animal operations: I. A review of enteric methane mitigation options. J. Anim. Sci..

[6] Beauchemin K.A., Ungerfeld E.M., Eckard R.J., Wang M. (2020). Review: Fifty Years of research on rumen methanogenesis: Lessons learned and future challenges for mitigation. Animal.

[7] Field J.A., Kortekaas S., Lettinga G. (1989). The tannin theory of methanogenic toxicity. Biol. Wastes.

[8] Tavendale M.H., Meagher L.P., Pacheco D., Walker N., Attwood G.T., Sivakumaran S. (2005). Methane production from in vitro rumen incubations with Lotus pedunculatus and Medicago sativa, and effects of extractable condensed tannin fractions on methanogenesis. Anim. Feed Sci. Technol..

[9] Jayanegara A., Leiber F., Kreuzer M. (2011). Meta-analysis of the relationship between dietary tannin level and methane formation in ruminants from in vivo and in vitro experiments. J. Anim. Physiol. Anim. Nutr..

[10] Díaz Carrasco J.M., Cabral C., Redondo L.M., Pin Viso N.D., Colombatto D., Farber M.D., Fernández Miyakawa M.E. (2017). Impact of chestnut and quebracho tannins on rumen microbiota of Bovines. Biomed. Res. Int..

[11] Makkar H.P., Becker K., Abel H., Szegletti C. (1995). Degradation of condensed tannins by rumen microbes exposed to quebracho tannins (QT) in Rumen Simulation Technique (RUSITEC) and effects of qt on fermentative processes in the RUSITEC. J. Sci. Food Agric..

[12] Bhatta R., Uyeno Y., Tajima K., Takenaka A., Yabumoto Y., Nonaka I., Enishi O., Kurihara M. (2009). Difference in the nature of tannins on in vitro ruminal methane and volatile fatty acid production and on methanogenic Archaea and protozoal populations. J. Dairy. Sci..

[13] Carulla J.E., Kreuzer M., Machmüller A., Hess H.D. (2005). Supplementation of Acacia mearnsii tannins decreases methanogenesis and urinary nitrogen in forage-fed sheep. Aust. J. Agric. Res..

[14] Aboagye I.A., Beauchemin K.A. (2019). Potential of molecular weight and structure of tannins to reduce methane emissions from ruminants: A Review. Animals.

[15] Kumar R., Singh M. (1984). Tannins: Their adverse role in ruminant nutrition. J. Agric. Food Chem..

[16] Min B.R., Barry T.N., Attwood G.T., McNabb W.C. (2003). The effect of condensed tannins on the nutrition and health of ruminants fed fresh temperate forages: A review. Anim. Feed Sci. Technol..

[17] Makkar H.P. (2003). Effects and fate of tannins in ruminant animals, adaptation to tannins, and strategies to overcome detrimental effects of feeding tannin-rich feeds. Small Rumin. Res..

[18] Huang Q., Liu X., Zhao G., Hu T., Wang Y. (2018). Potential and challenges of tannins as an alternative to in-feed antibiotics for farm animal production. Anim. Nutr..

[19] United States Code of Federal Regulations, 7 CFR § 205, December 21, 2000.

[20] Hristov A.N., Oh J., Giallongo F., Frederick T., Weeks H., Zimmerman P.R., Harper M.T., Hristova R.A., Zimmerman R.S., Branco A.F. (2015). The use of an automated system (GreenFeed) to monitor enteric methane and carbon dioxide emissions from ruminant animals. J. Vis. Exp..

[21] Gunter S.A., Bradford J.A. (2017). Technical note: Effect of bait delivery interval in an automated head-chamber system on respiration gas estimates when cattle are grazing rangeland. Prof. Anim. Sci..

[22] Gunter S.A., Duke S.E., Beck M.R. (2017). Measuring the respiratory gas exchange of grazing cattle using the GreenFeed emissions monitoring system. J. Anim. Sci..

[23] Huhtanen P., Ramin M., Hristov A.N. (2019). Enteric methane emission can be reliably measured by the GreenFeed monitoring unit. Livest. Sci..

[24] Velazco J.I., Mayer D.G., Zimmerman S., Hegarty R.S. (2016). Use of short-term breath measures to estimate daily methane production by cattle. Animal.

[25] Arthur P.F., Barchia I.M., Weber C., Bird-Gardiner T., Donoghue K.A., Herd R.M., Hegarty R.S. (2017). Optimizing test procedures for estimating daily methane and carbon dioxide emissions in cattle using short-term breath measures. J. Anim. Sci..

[26] Thompson L.R., Beck M.R., Gunter S.A., Williams G.D., Place S.E., Reuter R.R. (2019). An energy and monensin supplement reduces methane emission intensity of stocker cattle grazing winter wheat. Appl. Anim. Behav. Sci..

[27] Armstrong D., Browne R. (1994). The analysis of free radicals, lipid peroxides, antioxidant enzymes and compounds related to oxidative stress as applied to the Clinical Chemistry Laboratory. Adv. Exp. Med. Biol..

[28] Beck M.R., Gregorini P. (2020). How dietary diversity enhances hedonic and eudaimonic well-being in grazing ruminants. Front. Vet. Sci..

[29] Malmstrom B.G., Andreasson L.-E., Reinhammar B. (1975). Copper-containing oxidases and superoxide dismutase. Enzymes.

[30] Aquilano K., Baldelli S., Ciriolo M.R. (2014). Glutathione: New roles in redox signaling for an old antioxidant. Front. Pharmacol..

[31] Littell R.C., Henry P.R., Ammerman C.B. (1998). Statistical analysis of repeated measures data using SAS procedures. J. Anim. Sci..

[32] Ahlberg C.M., Allwardt K., Broocks A., Bruno K., McPhillips L., Taylor A., Krehbiel C.R., Calvo-Lorenzo M., Richards C.J., Place S.E. (2018). Test duration for water intake, ADG, and DMI in beef cattle1. J. Anim. Sci..

[33] Beauchemin K.A., McGinn S.M., Martinez T.F., McAllister T.A. (2007). Use of condensed tannin extract from quebracho trees to reduce methane emissions from cattle. J. Anim. Sci..

[34] Ebert P.J., Bailey E.A., Shreck A.L., Jennings J.S., Cole N.A. (2017). Effect of condensed tannin extract supplementation on growth performance, nitrogen balance, gas emissions, and energetic losses of beef steers. J. Anim. Sci..

[35] Piñeiro-Vázquez A.T., Jiménez-Ferrer G., Alayon-Gamboa J.A., Chay-Canul A.J., Ayala-Burgos A.J., Aguilar-Pérez C.F., Ku-Vera J.C. (2017). Effects of quebracho tannin extract on intake, digestibility, rumen fermentation, and methane production in crossbred heifers fed low-quality tropical grass. Trop. Anim. Health Prod..

[36] Johnson D., Almaraz M., Rudnick J., Parker L.E., Ostoja S.M., Khalsa S.D. (2023). Farmer adoption of climate-smart practices is driven by farm characteristics, information sources, and practice benefits and challenges. Sustainability.

[37] Johnson K.A., Johnson D.E. (1995). Methane emissions from cattle. J. Anim. Sci..

[38] Lipper L., Thornton P., Campbell B.M., Baedeker T., Braimoh A., Bwalya M., Caron P., Cattaneo A., Garrity D., Henry K. (2014). Climate-smart agriculture for food security. Nat. Clim. Chang..

[39] National Research Council (2001). Nutrient Requirements of Dairy Cattle.

[40] Ramana D. (2023). Climate Change Impacts and Innovative Adoption Options for Smart Animal-Agriculture. Impact of Climate Change on Livestock Health and Production.

[41] Liu H.W., Zhou D.W., Li K. (2013). Effect of chestnut tannins on performance and antioxidative status of transition dairy cows. J. Dairy Sci..

