# Correction: Schilling-Hazlett et al. On-Farm Methane Mitigation and Animal Health Assessment of a Commercially Available Tannin Supplement in Organic Dairy Heifers. *Animals* 2024, *14*, 9

**DOI:** 10.3390/ani14162424

**Published:** 2024-08-21

**Authors:** Ashley Schilling-Hazlett, Edward J. Raynor, Logan Thompson, Juan Velez, Sara Place, Kim Stackhouse-Lawson

**Affiliations:** 1 CSU AgNext, Department of Animal Sciences, Colorado State University, Fort Collins, CO 80523, USA; 2 Department of Animal Science and Industry, Kansas State University, Manhattan, KS 66502, USA; 3 Aurora Organic Dairy, Boulder, CO 80302, USA

**Text Correction**

There was an error in the original publication [1]. The methodology for determining average daily gain was misinterpreted.

A correction has been made to Materials and Methods, Sample Collection, Animal Body Weights:

This correction will result in omitting the sentence in Section 2.2.4 describing the use of linear regression to derive average daily gain.

 

2.2.4. Animal Body Weights

Heifers were weighed (unshrunk) on d −15, 0, 8, 15, 23, 29, 36, 43, and 45 before treatment feeding at 0700 h on a validated scale (Tru-Test AP600 platform and Tru-Test ID5000 scale indicator) using the methodologies described by Thompson et al. [26].

 

**Text Correction**

There was an error in the original publication. The methodology for determining average daily gain was misinterpreted, which will impact citations is subsequent sections.

A correction has been made to Materials and Methods, Statistical Analysis, Paragraph 1:

This correction will result in omitting the sentence in section describing the use of linear regression to derive average daily gain and omitting reference [32] and the description of using linear regression to derive average daily gain. With this correction, the order of some references will require ajdustment.

 

*2.3. Statistical Analysis*

JMP^®^ Pro (JMP^®^, v. 16.2.0. SAS Institute Inc., Cary, NC, USA, 1989–2021) software was used for initial data visualization and correlation analyses. Subsequent analysis of data was conducted using R© (R Core Team, 2021, v. 4.1.2) software. The data were analyzed using the gls() function within the R package nlme in which tannin treatment, TMR diet, treatment × TMR diet, and mean 7 d pre-trial CH4 (g/d) baseline were fixed factors, and an individual animal was a random intercept. A first-order autoregressive error structure was also included for repeated measures on each animal. The covariance structure that best fit the data was selected according to Schwartz’s Bayesian information criterion [31]. The model diagnostics included testing for normal distribution of the error residuals and homogeneity of variance. The assumptions were adequately held. Dependent variables were ADG, DMI, G:F (kg BW gain/kg DMI), daily CH4 production (g CH4/hd/d), CH4 as a percentage of gross energy (GE) intake (Ym), CH4 yield (MY; g CH4/kg DMI), CH4 emission intensity (EI; g of CH4/kg ADG), BUN (mg/dL), creatinine (mg/dL), MDA (μM), SOD (units/mL), and GSH (μM). Daily CH4 production was analyzed using the 7 d pretrial emissions rate as a covariate (i.e., an individual-specific baseline measurement of pretrial emissions) [20]. The effect of treatment was determined to be significant at α < 0.05 and a tendency between 0.05 < *p* ≤ 0.10. Sattherwaite’s approximation was used to calculate the effective degrees of freedom of a linear combination of independent sample variances. To examine a potential dose-response relationship for heifers consuming increasing levels of tannin, linear and quadratic effects were assessed using orthogonal contrasts [32].

 

**Error in Table**

In the original publication, there was a mistake in Table 3 and Table 4 as published. The authors slightly misinterpreted the methodology and results for emissions intensity (EI) and average daily gain (ADG) and have provided the corrected means and variance for EI and ADG in Table 3 and Table 4, respectively. The corrected results do not change the outcome of this study or the Discussion. The corrected Table 3 and Table 4 appear below. The authors state that the scientific conclusions are unaffected. This correction was approved by the Academic Editor. The original publication has also been updated.

## Figures and Tables

**Table 3:** Mean CH_4_ emission measurements for heifers supplemented with Silvafeed^®^ ByPro from d 0 to 45 fed in northeastern Colorado from November 2021 to January 2022.

	Control	Tannin	SE	*p*-Value ^1^	Treatment ^2^				SE	*p*-Value ^3^		
Parameter ^4^					CON	LOW	MED	HIG		Linear	Quadratic	TMR
n	5	15			5	5	5	5				
CH_4_ Production, g CH_4_/hd/d	146	145	5.2	0.88	146	145	149	142	5.2	0.56	0.52	0.002
Y_m_,%GE intake	6.7	6.8	0.01	0.92	6.7	7.7	6.8	5.7	0.01	0.13	0.21	0.01
MY, g CH_4_/kg DMI	23.4	23.6	2.2	0.91	23.4	27.1	23.9	19.9	2.2	0.13	0.21	0.01
EI, g CH_4_/kg ADG	109.9	141.8	30.3	0.30	109.9	148.2	126.9	150.1	26.8	0.38	0.79	0.004

^1^ Significance *p* < 0.05, ^2^ treatments for tannin supplementation at 0% (CON), 0.075% (LOW), 0.15% (MED), and 0.30% (HIG) of DMI, ^3^ significance *p* < 0.05, ^4^ methane (CH_4_) production, average daily gain (ADG), dry matter intake (DMI), CH_4_ as % of GE intake (Y_m_), CH_4_ yield (MY), CH_4_ emissions intensity (EI), feed efficiency (G:F).

**Table 4:** Mean performance measurements for heifers supplemented with Silvafeed^®^ ByPro from d 0 to 45 fed in northeastern Colorado from November 2021 to January 2022.

	Control	Tannin	SE	*p*-Value ^1^	Treatment ^2^				SE	*p*-Value ^3^		
Parameter ^4^					CON	LOW	MED	HIG		Linear	Quadratic	TMR
n	5	15			5	5	5	5				
ADG, kg BW gain/d	0.50	0.52	0.23	0.92	0.50	0.57	0.50	0.50	0.20	0.91	0.90	<0.001
FBW, kg	250	252	1.13	0.11	250	254	251	251	1.13	0.70	0.07	-
DMI, kg DMI/d	6.2	6.7	0.72	0.61	6.2	6.4	6.6	7.0	0.72	0.42	0.96	0.06
TMR, kg TMR DMI/d	4.9	5.3	0.72	0.66	4.9	5.0	5.2	5.6	0.72	0.45	0.93	0.07
SF, kg SF DMI/d	0.93	0.93	0.00	-	0.93	0.93	0.93	0.93	0.00	-	-	-
GF, kg GF DMI/d	0.34	0.40	0.03	0.07	0.34	0.44	0.38	0.40	0.03	0.46	0.27	0.02
G:F, ADG/DMI	0.10	0.09	0.03	0.66	0.10	0.11	0.08	0.08	0.03	0.46	0.94	<0.001

^1^ Significance *p* < 0.05, ^2^ treatments for tannin supplementation at 0% (CON), 0.075% (LOW), 0.15% (MED), and 0.30% (HIG) of DMI, ^3^ significance *p* < 0.05, ^4^ average daily gain (ADG), final body weight (FBW), dry matter intake (DMI), total mixed ration (TMR), sweet feed (SF), GreenFeed bait pellets (GF), feed efficiency (G:F).
